# Knowledge, Attitude, and Practice toward Youth-Friendly Reproductive Health Services among Mizan-Tepi University Students, South-Western Ethiopia

**DOI:** 10.1155/2022/2312407

**Published:** 2022-03-20

**Authors:** Samuel Getachew, Lema Abate, Abyot Asres, Abel Mandefro

**Affiliations:** ^1^Department of Biology, MSc.in Biomedical Sciences, College of Natural and Computational Sciences, Mizan-Tepi University, Tepi, Ethiopia; ^2^Department of Statistics, MSc. in Biostatistics, College of Natural and Computational Sciences, Mizan-Tepi University, Tepi, Ethiopia; ^3^Department of Public Health, College of Health Sciences, Mizan Tepi University, Mizan-Aman, Ethiopia; ^4^Department of Biology, BSc. in Applied Biology, College of Natural and Computational Sciences, Mizan-Tepi University, Tepi, Ethiopia

## Abstract

**Background:**

Youth-friendly reproductive health services are designed to meet the unique needs of young individuals. Nevertheless, in developing countries such as Ethiopia, knowledge, attitude, and utilization of these services are very limited. This study was designed to assess the knowledge, attitude, and utilization of youth-friendly health services and associated factors among students.

**Method:**

A public university-based cross-sectional study was conducted between October and January 2019. Participants were chosen from the target group using a simple random selection procedure. To find the factors linked to youth-friendly health services, researchers used descriptive analysis, the chi-square test, and a logistic regression model.

**Result:**

This study revealed that 237 (55.4%), 256 (59.8%), and 262 (61.2%) students had no knowledge, negative attitude, and not practiced youth-friendly reproductive health services, respectively. The binary logistic regression results revealed that male students were more likely to have knowledge and practice (AOR = 1.847; 95% CI: 1.124, 3.034; *p*=0.015) and (AOR = 1.821; 95% CI: 1.114, 2.975; *p*=0.017) respectively; however, they had less likely positive attitudes (AOR = 0.519; 95% CI: 0.315, 0.856; *p* value = 0.010) compared to female students. Students from primary and above educated families were more likely to have knowledge, attitude, and practice compared to students from uneducated families.

**Conclusion:**

Overall, students' knowledge, attitudes, and utilization of youth-friendly reproductive health services were poor. As a result, additional efforts, such as the availability of service providers and the improvement of facilities, as well as education linked to the service for young people and the allocation of appropriate service time, are required.

## 1. Background

Youth is a period of transition from childhood to adulthood, and adolescence (from ages 10 to 19 years) is the process of attaining sexual and reproductive maturity [1, 2]. Typical youth behaviors such as experimentation and risk-taking make youths more vulnerable to pregnancy and STDs. Therefore, provision of youth-friendly sexual and reproductive health services is a first step to ensure the psychological and biomedical needs of young individuals [3].

In 2002, the World Health Organization (WHO) created the adolescent-friendly health services model, which was later expanded to accommodate all young people's health requirements [4, 5]. Youth-friendly service (YFS) is one of the reproductive health services offered to young people in order to satisfy their needs. It includes sexual and reproductive health counseling, sexually transmitted infections (STIs), and voluntary counseling and testing (VCT) services [4, 6, 7], and these services should be able to effectively attract and meet the needs of young people in a comfortable and responsive manner, as well as successfully retain these young clients for long-term care [1, 8, 9].

Seventy-eight percent of youths live in developing countries, and 60% of them are from sub-Saharan Africa, where the prevalence of HIV, other sexually transmitted infections (STIs), and unwanted pregnancy among young people is found to be high [1, 8, 10]. In Ethiopia, young people and adolescents in the age group of 10 to 24 years constitute 22% of the total population, most of them live in rural areas and lack access and awareness to the reproductive health services [7, 11]. The Ethiopian Ministry of Health (EMOH) has developed a 10-year National Adolescent and Youth Reproductive Health Strategy (NAYRHS) in 2015, utilization of the services among youths of the country is still low and remains facing several challenges associated with traits related to adolescents' sexuality and their perception towards the YFS delivery centers.

According to research evidence from various sections of Ethiopia, many adolescents still lack access to health care and young people are at risk of reproductive health issues. YFRHS provision is limited in a few government (public) health institutions in metropolitan centers, according to recent studies from rural areas, and many youth are less aware, experienced, and comfortable in obtaining health care in their locations. Moreover, majority of the youth often lack basic YFRHS information and knowledge about the available services, and access to affordable and confidential services [11–15]. As a result, the majority of teenagers are at an increased risk of unintended pregnancy and pregnancy-related problems [16].

Moreover, reproductive tract infections (RTIs) including HIV/AIDS are other major health threats of young people of the country [7, 11, 12]. However, studies on the level of YFRHS utilization, knowledge and perceptions of youths towards YFRHS, and associated factors are very limited in Ethiopia. Thus, this study was carried out with the objective of assessing YFRHS and associated factors among the students of Mizan-Tepi University, Tepi campus, southwestern Ethiopia.

## 2. Methods

### 2.1. Study Setting

The study was conducted at Mizan-Tepi university Tepi campus which is situated in Tepi-611 kms away from the capital of Ethiopia. Tepi campus is located in Yeki-Woreda at a mean elevation of 1,097 meters above the sea level and at a latitude of 7°12'N and longitude of 35°27'E. Tepi is the largest settlement in Yeki Woreda, and its climatic condition is characterized by higher temperature, higher moisture content, and lower wind speed. The annual rain fall is 1547 with 22.4°C average temperature, and its altitude is 1400 m above the sea level.

Tepi campus is among the three campuses of Mizan-Tepi University (two campuses found in Mizan-Aman), and it was established in 2006GC. According to information of the student's and alumni directorate of Tepi campus, under College of Natural and computational sciences, College of Engineering Technology, and School of Computing and Informatics, there are 16 departments with a total of 5739 students, of whom 4188 are male [Personal communication].

### 2.2. Study Variables

The dependent variables in this study were knowledge, attitude, and practice of students toward YFRHS, while the explanatory variables were sex, age, region, religion, mother's education, father's education availability of YFRHS in local area, and source of information.

### 2.3. Study Design and Population

A public university-based cross-sectional analysis was performed to assess the knowledge, attitude, and practices of MTU-Tepi campus students towards youth-friendly services between October and January 2019. All students of MTU-Tepi campus under two colleges (College of Natural and Computational Sciences and College of Engineering and Technology) and one school (School of Computing and Informatics) were considered. All regular students in the Tepi campus selected as target population of this study. There is homogeneity among the study population, and in addition, the target population was known; therefore, the simple random sampling technique used to select the sample from target population by applying the lottery method.

### 2.4. Inclusion and Exclusion Criteria

All class attending regular and volunteer students to enroll in the study are included, whereas those seriously ill and not volunteer to participate were excluded from the study.

### 2.5. Sample Size Determination

The sample size was calculated using a single population proportion formula using the parameters: confidence level 95% (*Z*_*α*/2_=1.96), margin of error 5% (*d* = 0.05), and assuming the prevalence of having knowledge of YFHS 50% (*p*=0.5). Then, the sample size (*n*) was determined as follows:

(1) $$\begin{array}{c} n=\frac{{\left(Z_{\alpha /2}\right)}^{2}p\left(1-p\right)}{d^{2}},\;\\ n=\frac{{\left(1.96\right)}^{2}0.5\left(1-0.5\right)}{0.0025}=385. \end{array}$$

By considering 11% of none response rate, the sample size was calculated as 428.

### 2.6. Data Collection Procedure and Instrument

After acquiring consent and voluntariness from each respondent, basic sociodemographic and socioeconomic data, as well as sexual-related information, were obtained using a pretested, semistructured self-administered questionnaire.

### 2.7. Statistical Data Analysis

The collected data were cleared, coded, and analyzed using SPSS version 20. For statistical analysis, from descriptive statistics: frequency and percentage and from inferential statistics: chi-square and multiple logistic regression with odd ratio (OR) were applied to identify the significant factors. The predictor variables that were significant in the univariable analysis at the 25% (*p* value <0.25) level of significance were included in the multiple logistic regression analysis. The estimated odds ratios (OR) and 95% confidence intervals with *p* value less than 5% indicate that the variables are statistically significant in multivariable analysis.

### 2.8. Operational Definition of Terms

Youth is the period between childhood and adulthood which involves unique physiological, psychological, cognitive, social, and economic changes, describing individuals in the age range of 15 and 24 years. Youth-friendly health services are services that are considered as sociable, ideal, and suitable for providing solutions for the various health concerns of young people. Youth-friendly centers are any health facility that provides friendly services to the youth [4, 7, 17].

## 3. Results

### 3.1. Sociodemographic Characteristics of Study Participants

**Figure 1:**
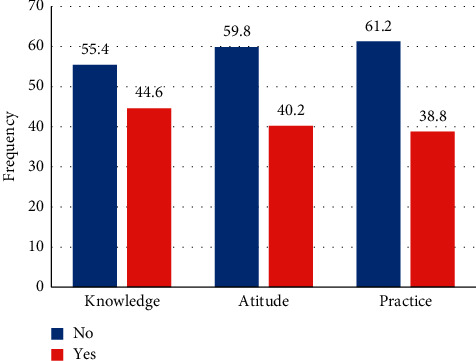
Prevalence of knowledge, attitude, and practice towards YFRHS.

This study revealed that 237 (55.4%) of students had no knowledge about YFHRS, 256 (59.8%) of students had negative attitude towards YFHRS, and 262 (61.2%) of students did not practiced any kinds of YFHRS (Figure 1). From a total of 428 students enrolled in the study, 258 (60.30%) were female and 209 (48.80%) were in the age group of 21-22 years. Most of the students are from Oromia 105 (24.5%), Addis Ababa 71 (16.6%), and SNNPRS 70 (16.4%). Educational background of 145 (33.9%) and 96 (22.4%) of the mother and father of students was uneducated, while 102 (23.8%) and 80 (18.7%) of their parents have attended primary school, respectively. Most of the students in the study were followers of Orthodox 126 (29.4%) and Protestant religions 117 (27.3%) (Table 1).

### 3.2. Knowledge, Attitude, and Practice of Respondents

From the total participants, 237 (55.4%) of the students have no knowledge of the youth-friendly health service (YFHS) (Table 2) and never heard of any information about YFRHS. The majority (220 (51.4%)) of them reported having no any YFRHS in their surroundings, while 58 (13.6%) do not know whether there is the service or not. For 131 (30.6%) and 151 (35.3%) of students, their friends and school teachers were the major sources of information about YFHS, respectively. The role of media as a source of information about YFHS is limited to about 21% (4.7% from newspaper and 17.8% from different media). Two hundred forty-eight (57.9%) of the respondents answered that family planning and contraception were services provided at YFRHS centers (Table 2).

About 53% of the respondents believe that provision of contraception for a young person is obligatory and 53.3% argue that every young person should aware of YFRHS. From total students, 72.4% of the respondents disagree that female students are not the only one to use YFRHS (Table 3). Out of 428 respondents, 57.9%, 59.3%, and 36.4% had never been practiced services related to VCT, family planning and contraception, and treatment of STIs at YFRHS, respectively (Table 4).

### 3.3. Association of Knowledge of Students and Explanatory Variables

The results of the chi-square test of association revealed that sex of the student, age of the student, mother education status, father education status, availability of service providers in the student's area, and source of information had statistically significant association with knowledge of students toward YFHS, as summarized in Table 5. The region and religion of students had no any statistically significant relationship.

### 3.4. Knowledge, Attitude, and Practices and Associated Factors of Youth-Friendly Health Services among Respondents

The results of binary logistic regression analysis using the forward variable selection method of the parameter revealed that sex, age, mother education, father education, availability of YFRS in the area, source of information about YFRHS, and religion had statistically significant effect on the knowledge, attitude, and practices of students toward YFHS. In this regard, male students were more likely to have knowledge and practice (AOR = 1.847; 95% CI: 1.124, 3.034; *p*=0.015) and (AOR = 1.821; 95% CI: 1.114, 2.975; *p*=0.017) respectively; however, they had less likely positive attitudes (AOR = 0.519; 95% CI: 0.315, 0.856; *p* = 0.010) compared to female students. Students whose age ranges 21-22 years were 2.732 times and 23-24 years were 4.345 times more likely to have knowledge compared to 18–20-year age groups (AOR = 2.732; 95% CI: 1.405, 5.313; *p*=0.003) and (AOR = 4.345; 95% CI: 1.742, 7.168; *p*=0.002, respectively). Students in age group 21-22 years were 2.275 times more likely to practice YFRHS compared to respondents in the age group 18–20 years. Students from Tigiray and Oromia regions were less likely to have knowledge about YFRHS (AOR = 0.314; 95% CI: 0.110, 0.896; *p*=0.030) and (AOR = 0.452; 95% CI: 0.207, 0.986; *p*=0.046) compared to students from Addis Ababa city administration (Table 6).

The odds ratios for students from primary school educated mothers were (AOR = 1.274; 95% CI: 1.139, 2.541; *p*=0.001), (AOR = 3.087; 95% CI: 1.557, 6.121; *p*=0.001), and (AOR = 2.541; 95% CI: 1.160, 5.569, *p*=0.001), which implies that students from primary school educated mothers were 1.274, 3.087, and 2.541 times more likely to have knowledge, positive attitude, and practice of YFHS than uneducated mothers, respectively. Students from secondary school educated mothers had odd ratios (AOR = 1.413; 95% CI: 1.185, 3.919; *p*=0.030), (AOR = 2.485; 95% CI: 1.108, 5.571, *p*=0.027), and (AOR = 3.279; 95% CI: 1.516, 7.094; *p*=0.019), which implies that students from secondary school educated mothers were 1.413, 2.485, and 3.279 times more likely to have the knowledge, positive attitude, and practices towards YFRHS than from uneducated mothers, respectively.

The odds ratio for students from fathers of different educational backgrounds had statistically significant effect on knowledge, attitude, and practicing of YFRHS. As summarized in Table 6, students whose fathers were educated primary and above had odds ratio greater than one, and this implies that those students were more likely to have knowledge and positive attitude of YFRHS compared to from uneducated father. Students having YFRHS in their surroundings were more likely to have knowledge and practicing experience (AOR = 3.230; 95% CI: 1.908, 5.469; *p*=0.001) and (AOR = 2.475; 1.469, 4.168; *p*=0.001) compared to students not having any service in their surroundings, respectively (Table 6).

Students who get information from newspaper and media were 0.119 and 0.288 times less likely to had knowledge (AOR = 0.119; 95% CI: 0.027, 0.529; *p*=0.005) and (AOR = 0.288; 95% CI: 0.112, 0.738; *p*=0.009) compared to students who get information from their parents. The odds ratio for students who get information from newspaper (AOR = 7.776; 95% CI: 1.764, 34.290; *p*=0.007) and media (AOR = 5.177; 95% CI: 1.944, 13.787; *p*=0.001), which implies that students who get information from newspaper and media were more likely to have positive attitude of YFHS than students who get information from their parents (Table 6).

## 4. Discussions

Out of the students of Mizan-Tepi University Tepi campus enrolled in the study, 220 (51.4%) reported having no access to any YFRHS in their locality, and this was comparable with previous studies conducted in Jimma, 47.1% [18]. However, it was much higher than a study conducted in Bahir Dar, 13.6% [19]; Adiss Ababa, 40% [20]; and community based study in Jimma 41% [18] and Harar 36% [21]. For 131 (30.6%) and 151 (35.3%) of the respondents, their friends and school teachers were the major sources of information about YFRHS, respectively, and this is quite similar with findings from Mekelle Town and Bahir Dar that reported the media outlets as a source of information are for 35.5% of the students [19]. Whereas, finding of this study is in contrast to studies done in Nigeria [22], Tanazania [23], and Ghana [1] that indicates parents, close friends, and peers of youths are the most common sources of information.

In this study, about 21% of participants got information from different medias, and this was in agreement with a study done in Harar that showed media as source of information for 22.8% participants [24]. However, this finding is less than a study in Bahir Dar and Mekelle (35.5%) [15, 19]. The majority (55.4%) of respondents lack basic information and knowledge of YFHRS, and this was similar with a study in Meda Wolabu University students [16]. However, it was found much less than a research from Hadiya Ethiopia (78.5%) [25]. On the other hand, 27.6% of the respondents believed that female students are the only one to use youth-friendly health service, and this agrees with research studies done in Moldova [9].

Of a total of 428 participants, only 49% had utilized YFRHS, which is higher than a study report from Bahir Dar (32%) [19]. Treatment of STI services (63.6%) and VCT (42.1%) were the most utilized services by the study cases. However, family planning and contraception (40.7%) was a less utilized service at YFRHS center. This was in contrast to the finding in Hadiya Zone, Ethiopia [25], that reported VCT 343 (68.9%) and contraception 321 (64.5%) as the most utilized YFRHS. One of the most reported reasons by the students for not utilizing the services was the unavailability of the service in their locality (51.1%) and less availability of services even in some of the areas where the service provider is available, and this was also reported in West Gojjam Zone, Ethiopia [13]. Other study from Bahir Dar indicated inconvenience hours and fear of being seen by parents, colleagues, neighbors, or other people as roadblocks in utilizing reproductive health services [19]. Among the respondents, condom 195 (45.6%) and emergency pills 169 (39.5%) are the most commonly used contraceptive methods. This finding was supported with other studies conducted in different parts of the Ethiopia [7, 13, 14] and beyond like South Africa and Tanzania [23, 26, 27]. In addition, this requires a great attention of both governmental and nongovernmental organizations working in provision of YFRHS across parts of Ethiopia. On chi-square test analysis, the factors that were found to be significantly associated with knowledge of students toward YFRHS were sex, age, mother's education and father's educational status, availability of any YFRH service in the respondents' area, and source of information. This was in agreement with studies conducted in Bale [2] and Wollisso [28].

Male students had more knowledge and practicing experience than female student, and this is in line with studies done in South Gondar [29], Gujarat India [30], and China [31]. This might be lack of awareness given for female students on YFRHS or lack of service giving facility in their surroundings nearby. However, it is opposing to the study done in East Gojjam zone, Ethiopia [32]. Students in age group 21-22 years were 2.732 times and in age group 23-24 years were 4.345 times more likely to have knowledge compared to student in the age group of 18–20 years, respectively, Moreover, students in the age group of 21-22 years were 2.275 times more likely experienced YFHS compared to students in the age group of 18–20 years. This study is in agreement with studies conducted in Bahir Dari [19], Jimma city [18], India [33], and East Gojjam [32]. Following the growth of students' in age, there is a subsequent increase in having knowledge about YFHS, this might be due to information gap about the service on time. However, it contradicts with study done using multicountry data analysis in China [31].

Students whose permanent residence was Tigiray and Oromia regions had less knowledge about YFHS compared to Addis Ababa city administration. This could be due to student from urban who had more YFHS offering facility access than students from rural parts of the country. As Addis Ababa is the capital city of Ethiopia and also there are many governmental and nongovernmental health facilities, mother and father education status had statistically significant effect on the knowledge, attitude, and practice of students toward YFHS. As shown in this finding, when the mother and father education level increased, the knowledge, attitude, and practice of students toward YFRHS also increased. This study is in line with study conducted in Awabel district northwest Ethiopia [34], and it is contradicting with the study done in south Gondar [29].

Students who had YFRHS access in their surroundings were 3.23 times more likely to have knowledge and 2.475 times more likely practicing experience about YFRHS compared to students who had no any service in their surroundings, respectively, this study is consistent with studies done in Hadiya Zone, Ethiopia [25], and Kenya [35]. Having this information, it can be expected as youths becoming more familiar with the service settings as well as types of services offered, and they will freely adopt to what type of service they have to use. Students who get information about YFRHS from their parents are more likely to have knowledge compared to students who get from different media. This finding agreed with study conducted in Awabel district northwest Ethiopia [34]. Students who get information from newspaper were 7.776 times more likely to have positive attitude compared to students who get information from their parents. This study is consistent with study conducted in Jimma city [18]. Participants who get information from media were 5.177 times more likely to have positive attitudes compared to students who get information from parents. According to practicing experience of YFRHS, this study revealed that there is opposite outcomes for practice and attitudes based on the source of information. This might be due to students who had information about the YFRHS, but no any services given for them.

## 5. Conclusion

The findings of this study demonstrated that the respondents' knowledge, attitude, and use of youth-friendly reproductive health services were insufficient. Above half of participants 237 (55.4%) had no knowledge, 256 (59.8%) had negative attitude, and 262 (61.2%) were not practiced youth-friendly reproductive health services. Students from secondary and above educated Mothers were more likely to have knowledge and practice YFRH services. Most of respondents had no awareness about the service, and it was a big problem to the student to use the service. This study revealed that male participants were more likely to have knowledge and practice toward YFRH services compared to female; therefore, it needs more attention to be given for awareness about the services.

### 5.1. Recommendations

Most of participants had no knowledge, negative attitude, and not practiced the services; therefore, all concerned bodies must put in much more effort in designing and implementing appropriate information provision through the use of media outlets, as well as incorporating into the curriculum education schools and universities, in order to influence youth-friendly service knowledge, attitudes, and utilization. Furthermore, both governmental and nongovernmental groups should work together to improve the quality and accessibility of YFRHS in order to meet the needs of young people.

## Figures and Tables

**Table 1:** Sociodemographic characteristics and family background of students, 2019 (*n* = 428).

Variables	Categories	Frequency	Percent
Sex	Female	258	60.3
	Male	170	39.7

Age (years)	18–20	71	16.6
	21–22	209	48.8
	23–24	148	34.6

Region	Addis Ababa	71	16.6
	Tigiray	28	6.5
	Amhara	55	12.9
	SNNPRS	70	16.4
	Oromia	105	24.5
	Gambela	30	7.0
	Other	69	16.1

Religion	Orthodox	126	29.4
	Protestant	117	27.3
	Catholic	64	15.0
	Muslim	90	21.0
	Other	31	7.2

Mother's educational status	Uneducated	145	33.9
	Primary	102	23.8
	Secondary	71	16.6
	Diploma	41	9.6
	Degree & above	69	16.1

Father's educational status	Uneducated	96	22.4
	Primary	80	18.7
	Secondary	112	26.2
	Diploma	70	16.4
	Degree and above	70	16.4

**Table 2:** Knowledge of youth-friendly service among students of MTU-Tepi campus, 2019 (*n* = 428).

Characteristics		Frequency	Percent
Have you ever heard any information about YFRHS?	No	237	55.4
	Yes	191	44.6

Is there any YFRH service in your area?	Yes	150	35.0
	No	220	51.4
	I do not know	58	13.6

Source of information about YFRHS?	Friends	131	30.6
	School teachers	151	35.3
	Media	96	22.4
	Health institutions	56	13.1

What are the services given at YFRHS providers?	Family planning and contraception	248	57.9
	VCT service	155	36.2
	Treatment of STIs	23	5.8

**Table 3:** Attitudes of students towards youth-friendly health service (*n* = 428).

No.	Characteristics	SD^*∗*^	%	DA	%	Neutral	%	Agree	%	SA^*∗*^	%
1	Unmarried youth have to visit YFRHS centers	38	8.9	88	20.6	96	22.4	172	40.2	34	7.9
2	The provision of contraception for young people is obligatory	28	6.5	110	25.7	62	14.5	208	48.6	20	4.7
3	Every young person should aware of YFRHS	6	1.4	58	13.6	108	25.2	144	33.6	112	26.2
4	Only females should use youth-friendly health service	62	14.5	248	57.9	54	12.6	50	11.7	14	3.3
5	Each unmarried young female pregnant can use safe abortion care	50	11.7	190	44.4	88	20.6	90	21.0	10	2.3
6	Every youth should use VCT service	60	14	134	31.3	94	22.0	126	29.4	14	3.3
7	Provision of YFRHS in the health institution are adequate	322	75.2	57	13.3	49	11.4	—		—	
8	Provision of YFRHS in both public and private health facilities should be free	—	—	—	—	36	8.4	—	—	392	91.6

^*∗*^SD: strongly disagree, DA: disagree, and SA: strongly agree.

**Table 4:** Practice of utilization of young-friendly reproductive health services among students of MTU-Tepi campus, 2019 (*n* = 428).

Characteristics	Categories	Frequency	Percent
Family planning and contraception	No	254	59.3
	Yes	174	40.7

Voluntary counseling test (VCT)	No	248	57.9
	Yes	180	42.1

Treatment of STI	No	156	36.4
	Yes	272	63.6

**Table 5:** Association of knowledge of YFHS among students and explanatory variables.

Variables	Chi-square test	Degree freedom	*p* value
Sex of the student	2.614	1	0.010^*∗*^
Age of the student	19.592	2	0.001^*∗*^
Region of the student	6.546	6	0.365
Mother's educational status	9.167	4	0.007^*∗*^
Father's educational status	16.199	4	0.003^*∗*^
Any service in the area	30.105	2	0.001^*∗*^
Source of information	33.546	4	0.001^*∗*^
Religion of the student	5.753	4	0.218

**Table 6:** Multiple logistic regression results of knowledge, attitude, and practice of students toward YFRHS.

Variables	Categories	Knowledge		Attitude		Practice	
		AOR (95% CI)	*p* value	AOR (95% CI)	*p* value	AOR (95% CI)	*p* value
Sex	Female	Ref.					
	Male	1.847 (1.124–3.034)	0.015^*∗*^	0.519 (0.315–0.856)	0.010^*∗*^	1.821 (1.114–2.975)	0.017^*∗*^

Age (years)	18–20	Ref.					
	21–22	2.732 (1.405–5.313)	0.003^*∗*^	0.418 (0.212–0.827)	0.012^*∗*^	2.275 (1.164–4.447)	0.016^*∗*^
	23–24	4.345 (1.742–7.168)	0.002^*∗*^	1.260 (0.611–2.599)	0.531	0.901 (0.443–1.836)	0.775

Region	Addis Ababa	Ref.					
	Tigiray	0.314 (0.110–0.896)	0.030^*∗*^	1.561 (0.520–4.686)	0.427	0.498 (0.167–1.482)	0.210
	Amhara	1.210 (0.550–2.664)	0.635	1.043 (0.473–2.302)	0.916	0.779 (0.360–1.687)	0.527
	SNNPR	0.753 (0.358–1.582)	0.453	1.104 (0.519–2.349)	0.797	0.646 (0.305–1.365)	0.252
	Oromia	0.452 (0.207–0.986)	0.046^*∗*^	1.900 (0.860–4.195)	0.112	0.495 (0.228–1.073)	0.075
	Gambela	0.667 (0.229–1.941)	0.457	1.293 (0.440–3.801)	0.640	0.699 (0.246–1.987)	0.501
	Other	0.812 (0.393–1.676)	0.573	0.967 (0.465–2.011)	0.928	0.799 (0.387–1.650)	0.544

Mother education status	Uneducated	Ref.					
	Primary	1.274 (1.139–2.541)	0.001^*∗*^	3.087 (1.557–6.121)	0.001^*∗*^	2.541 (1.160–5.569)	0.001^*∗*^
	Secondary	1.413 (1.185–3.919)	0.030^*∗*^	2.485 (1.108–5.571)	0.027^*∗*^	3.279 (1.516–7.094)	0.019^*∗*^
	Diploma	1.085 (0.398–2.957)	0.873	0.805 (0.294–2.208)	0.674	2.451 (1.034–5.813)	0.510
	Degree and above	0.678 (0.311–1.477)	0.328	1.348 (0.617–2.944)	0.454	7.125 (2.867–17.710)	0.595

Father education status	Uneducated	Ref.					
	Primary	2.605 (1.191–5.699)	0.017^*∗*^	0.574 (0.262–1.256)	0.165	2.541 [1.160–5.569)	0.020^*∗*^
	Secondary	3.544 (1.643–7.648)	0.001^*∗*^	0.318 (0.147–0.689)	0.004^*∗*^	3.279 [1.516–7.094)	0.003^*∗*^
	Diploma	2.725 (1.138–6.523)	0.024^*∗*^	0.428 (0.179–1.024)	0.057	2.451 [1.034–5.813)	0.042^*∗*^
	Degree and above	8.308 (3.299–20.923)	0.001^*∗*^	0.135 (0.053–0.341)	0.001^*∗*^	7.125 [2.867–17.710)	0.001^*∗*^

Any service in area	No	Ref.					
	Yes	3.230 (1.908–5.469)	0.001^*∗*^	0.392 (0.230–0.668)	0.001^*∗*^	2.475 (1.469–4.168)	0.001^*∗*^
	I do not know	2.559 (1.216–5.385)	0.013^*∗*^	0.345 (0.162–0.733)	0.006^*∗*^	1.476 (0.706–3.086)	0.301

Source of information	Parent	Ref.					
	Friend	0.927 (0.430–1.995)	0.846	1.733 (0.805–3.730)	0.160	0.738 (0.346–1.572)	0.431
	Teacher	1.657 (0.774–3.544)	0.193	0.663 (0.312–1.408)	0.285	1.691 (0.802–3.565)	0.168
	Newspaper	0.119 (0.027–0.529)	0.005^*∗*^	7.776 (1.764–34.290)	0.007^*∗*^	0.151 (0.034–0.668)	0.013^*∗*^
	Media	0.288 (0.112–0.738)	0.009^*∗*^	5.177 (1.944–13.787)	0.001^*∗*^	0.305 (0.119–0.785)	0.014^*∗*^

Religion	Orthodox	Ref.					
	Protestant	0.981 (0.527–1.823)	0.951	1.455 (0.781–2.711)	0.237	0.936 (0.508–1.724)	0.832
	Catholic	0.713 (0.333–1.527)	0.384	1.696 (0.790–3.639)	0.175	0.728 (0.341–1.554)	0.412
	Muslim	0.807 (0.404–1.609)	0.542	1.590 (0.792–3.193)	0.192	0.796 (0.401–1.579)	0.514
	Other	0.314 (0.110–0.896)	0.030^*∗*^	7.582 (2.318–24.796)	0.001^*∗*^	0.197 (0.062–0.624)	0.006^*∗*^

Ref.: reference categories, ^*∗*^significant variables at the 5% level of significance, and AOR: adjusted odds ratio.

## Data Availability

The datasets used in this study are available from the corresponding author on reasonable request.

## References

[1] Anokye-Mensah S. (2019). Knowledge, attitudes and perceptions of youth-friendly health services among adolescents in the Ashaiman district of Ghana. https://ugspace.ug.edu.gh/handle/123456789/35105.

[2] Kerbo A. A., Tefera T. B., Kuti K. A., Nur R. A. (2018). Youth friendly sexual and reproductive health services utilization and associated factors in Bale zone of Ethiopia: a community based cross sectional study. *Journal of Womens Health and Reproductive Medicine*.

[3] Senderowitz J. (1999). *Making Reproductive Health Services Youth Friendly*.

[4] World Health Organization (2003). *Adolescent Friendly Health Services: An Agenda for Change*.

[5] United Nations (2012). *World Population Monitoring: Adolescents and Youth–A Concise Report*.

[6] Wegelin-Schuringa M., Miedema E., van der Kwaak A., Karen‘t Hooft H. O. (2014). Youth friendly health services in multiple perspectives. https://www.Sharenetinternational.Org/Sites/Default/Files/Publication_file/YouthFriendlyHealthServicesinMultiplePerspectives.Pdf.

[7] Feleke S. A., Koye D. N., Demssie A. F., Mengesha Z. B. (2013). Reproductive health service utilization and associated factors among adolescents (15–19 years old) in Gondar town, Northwest Ethiopia. *BMC Health Services Research*.

[8] Koon A. D., Goudge J., Norris S. A. (2014). Considerations for linking south Africa’s youth-friendly services to its community health worker programme. *South African Journal of Child Health*.

[9] Carai S., Bivol S., Chandra-Mouli V. (2015). Assessing youth-friendly-health-services and supporting planning in the republic of Moldova. *Reproductive Health*.

[10] Mulugeta B., Girma M., Kejela G., Meskel F. G., Andarge E., Zerihun E. (2019). Assessment of youth-friendly service quality and associated factors at public health facilities in southern Ethiopia: a facility-based cross-sectional study. *BioMed Research International*.

[11] Liddon N., Steiner R. J., Martinez G. M. (2018). Provider communication with adolescent and young females during sexual and reproductive health visits: findings from the 2011–2015 national survey of family growth. *Contraception*.

[12] Muntean N., Kereta W., Mitchell K. R. (2015). Addressing the sexual and reproductive health needs of young people in Ethiopia: an analysis of the current situation. *African Journal of Reproductive Health*.

[13] Munea A. M., Alene G. D., Debelew G. T. (2020). Quality of youth friendly sexual and reproductive health services in west Gojjam zone, north west Ethiopia: with special reference to the application of the donabedian model. *BMC Health Services Research*.

[14] Yemaneh Y., Sahile E., Alehegn A., Girma A., Robles C. (2017). Assessment of the proportion and associated factors of episiotomy at public health institutions of Axum town, Tigray region, north Ethiopia, 2016. *Critical Care Obstetrics and Gynecology.*.

[15] Kahsay K., Berhe S., Alemayehu M. (2016). Utilization of youth friendly services and associated factors in Mekelle town, Tigray, northern Ethiopia. *International Journal of Therapeutic Applications*.

[16] Mengistu T. S., Melku A. T. (2013). Sexual and reproductive health problems and service needs of university students in south east Ethiopia: exploratory qualitative study. *Science Journal of Public Health*.

[17] Bearinger L. H., Sieving R. E., Ferguson J., Sharma V. (2007). Global perspectives on the sexual and reproductive health of adolescents: patterns, prevention, and potential. *Lancet*.

[18] Tegegn A., Gelaw Y. (2009). Adolescent reproductive health services in jimma city: accessibility and utilization. *Ethiopian Journal of Health Sciences*.

[19] Abebe M., Awoke W. (2014). Utilization of youth reproductive health services and associated factors among high school students in Bahir Dar, Amhara regional state, Ethiopia. *Open Journal of Epidemiology*.

[20] Habte D., Deyessa N., Davey G. (2006). Assessment of the utilization of pre-marital HIV testing services and shabbir ismael determinants of VCT in Addis Ababa, 2003. *Ethiopian Journal of Health Development*.

[21] Motuma A., Syre T., Egata G., Kenay A. (2016). Utilization of youth friendly services and associated factors among youth in Harar town, east Ethiopia: a mixed method study. *BMC Health Services Research*.

[22] Wright K. O., Oluwole E., Adeniran A., Kuyinu Y., Goodman O., Odusanya O. (2016). Youth friendly health services in a rural community of Lagos, Nigeria: are the youths receptive?. *International Journal of Adolescent Medicine and Health*.

[23] Alliance A. Y., Finder P. (2003). *Youth Friendly Sexual and Reproductive Health Services: An Assessment of Facilities*.

[24] Motuma A. (2012). Youth-friendly health services utilization and factors in Harar, Ethiopia. *Harar Bulletin of Health Sciences*.

[25] Helamo D., Kusheta S., Bancha B., Habtu Y., Yohannes S. (2017). Utilization and factors affecting adolescents and youth friendly reproductive health services among secondary school students in Hadiya zone, southern nations, nationalities and peoples region, Ethiopia. *International Journal of Public Health & Safety*.

[26] Geary R. S., Webb E. L., Clarke L., Norris S. A. (2015). Evaluating youth-friendly health services: young people’s perspectives from a simulated client study in urban south Africa. *Global Health Action*.

[27] Chilinda I., Hourahane G., Pindani M., Chitsulo C., Maluwa A. (2014). Attitude of health care providers towards adolescent sexual and reproductive health services in developing countries: a systematic review. *Health*.

[28] Mekonnen Y., Demissie T., Ababa A. (2005). *Kap Study on Hiv/Aids Among Adolescents and Assessment of Available Hiv/Aids Related Services in Wolliso Impact Area*.

[29] Abate A. T., Ayisa A. A., Tesfamichael G. (2019). Reproductive health services utilization and its associated factors among secondary school youths in Woreta town, south Gondar, north west Ethiopia: a cross sectional study. *BMC Research Notes*.

[30] Kotecha P. V., Patel S., Baxi R. K. (2009). Reproductive health awareness among rural school going adolescents of Vadodara district. *Indian Journal of Sexually Transmitted Diseases and AIDS*.

[31] Rani M., Lule E. (2004). Exploring the socioeconomic dimension of adolescent reproductive health: a multicountry analysis. *International Family Planning Perspectives*.

[32] Abajobir A. A., Seme A. (2014). Reproductive health knowledge and services utilization among rural adolescents in east Gojjam zone, Ethiopia: a community-based cross-sectional study. *BMC Health Services Research*.

[33] Barkat A., Majid M. (2003). *Adolescent Reproductive Health in Bangladesh: Status, Policies, Programmes and Issues*.

[34] Ayehu A., Kassaw T., Hailu G. (2016). Level of young people sexual and reproductive health service utilization and its associated factors among young people in Awabel District, Northwest Ethiopia. *PLoS One*.

[35] Ontiri K. K. (2015). Factors influencing utilization of reproductive health services amongst young people in rift valley provincial hospital, nakuru county-kenya. https://hdl.handle.net/11295/90160.

[36] Zewudie S. G., Adulo L. A., Sirna A. M., Asres A. (2020). *Youth Friendly Reproductive Health Services Among Students of Mizan-Tepi University, South-western Ethiopia*.

